# High-risk HPV infection after five years in a population-based cohort of Chilean women

**DOI:** 10.1186/1750-9378-6-21

**Published:** 2011-11-16

**Authors:** Catterina Ferreccio, Vanessa Van De Wyngard, Fabiola Olcay, M Angélica Domínguez, Klaus Puschel, Alejandro H Corvalán, Silvia Franceschi, Peter JF Snijders

**Affiliations:** 1 División de Salud Pública y Medicina Familiar, Escuela de Medicina Pontificia Universidad Católica de Chile, Marcoleta 434, Santiago 8330073, Chile; 2 Centro de Salud Familiar Santo Tomás, Santo Tomás 0987, Santiago 8830001, Chile; 3 International Agency for Research on Cancer, 150 cours Albert Thomas, 69372 Lyon cedex 08, France; 4 Department of Pathology, VU University Medical Center, De Boelelaan 1117, 1081 HV Amsterdam, The Netherlands

## Abstract

**Background:**

The need to review cervical cancer prevention strategies has been triggered by the availability of new prevention tools linked to human papillomavirus (HPV): vaccines and screening tests. To consider these innovations, information on HPV type distribution and natural history is necessary. This is a five-year follow-up study of gynecological high-risk (HR) HPV infection among a Chilean population-based cohort of women.

**Findings:**

A population-based random sample of 969 women from Santiago, Chile aged 17 years or older was enrolled in 2001 and revisited in 2006. At both visits they answered a survey on demographics and sexual history and provided a cervical sample for HPV DNA detection (GP5+/6+ primer-mediated PCR and Reverse line blot genotyping). Follow-up was completed by 576 (59.4%) women; 45 (4.6%) refused participation; most losses to follow-up were women who were unreachable, no longer eligible or had missing samples. HR-HPV prevalence increased by 43%. Incidence was highest in women < 20 years of age (19.4%) and lowest in women > 70 (0%); it was three times higher among women HR-HPV positive versus HPV negative at baseline (25.5% and 8.3%; OR 3.8, 95% CI 1.8-8.0). Type-specific persistence was 35.3%; it increased with age, from 0% in women < 30 years of age to 100% in women > 70. An enrollment Pap result ASCUS or worse was the only risk factor for being HR-HPV positive at both visits.

**Conclusions:**

HR-HPV prevalence increased in the study population. All HR-HPV infections in women < 30 years old cleared, supporting the current recommendation of HR-HPV screening for women > 30 years.

## Background

Most research on the natural history of human papillomavirus (HPV) infection and its progression towards cancer has been conducted in developed areas. In Chile, a middle developing country, the only two population-based studies to date analyzed the prevalence of HPV at one point in time [1,2]. Chile has a well-organized nationwide cervical cancer prevention program based on Papanicolaou screening, which, after an initial impact on cervical cancer mortality, has not produced a significant decrease in the last decade [3]. Moreover, Chile currently has a high socioeconomic status differential in cervical cancer mortality (C. Ferreccio, unpublished observations). The need to review prevention programs has been triggered by the availability of two new effective prevention tools linked to HPV: vaccines and screening tests. To consider these innovations, countries should have baseline information on HPV type distribution in their populations, as well as knowledge of how these infections evolve.

## Methods

In 2001 we studied the prevalence of gynecological HPV among a population-based sample of 969 women 17 years of age and older, from a low socio-economic urban area of Santiago. Results and detailed description of enrollment and data collection procedures were previously reported [1]. Five years later we re-examined these women with the objectives of measuring high-risk (HR) HPV prevalence at this second point in time, and assessing rates and risk factors of HR-HPV persistence. We attempted to contact these women for follow-up by home visits: 182 were not reachable, 45 declined participation, 22 were no longer eligible (pregnancy, hysterectomy, recent surgery, death) and 144 were excluded due to missing cervical samples. Therefore, data for the present analysis was available for 576 women (59.4%). At enrollment and follow-up, participants signed an informed consent form and completed a questionnaire on demographic and behavioral characteristics.

Participants attended a health center where a midwife collected cervical samples for HPV testing. An Ayre spatula was used to obtain exfoliated cervical cells and then placed in a tube with PBS; additionally, a spatula and cytobrush used for a Pap smear obtained at the same visit were washed in this tube. In both 2001 and 2006, HPV DNA testing was performed at the Department of Pathology of the VU University Medical Center in Amsterdam, the Netherlands. Specimens were tested for the presence of HPV DNA using a GP5+/6+ primer-mediated PCR and enzyme immune assay. Reverse line blot genotyping was used to type for 44 HPV genotypes, 14 HR-HPV (16, 18, 31, 33, 35, 39, 45, 51, 52, 56, 58, 59, 66, 68) [4].

Multivariate comparison of participants and non-participants in the follow-up examination was performed using logistic regression to identify predictors of participation. Prevalence of HR-HPV infection in 2001 and 2006 was compared, using prevalence rate ratios. Risk factors for HR-HPV incidence and persistence (age, marital status, schooling, number of children, age of first sexual intercourse, lifetime number of sexual partners, high-risk sexual partner, condom use, history of sexually transmitted disease, hormonal contraception, smoking, history of previous Pap tests, and an abnormal baseline Pap test) were examined with univariate analysis; variables significant at *P *value < 0.2 were then entered in multivariate models. Sexual behavioral changes in the study period were explored as risk factors for HR-HPV incidence. Statistical analyses were performed using SPSS version 17 for Windows.

## Results

Sociodemographic characteristics of the study sample are presented in Table 1. Because it was not initially designed as a prospective study, we could only recontact 59% of the original cohort; losses to follow-up were mainly due to changes in address (182) or loss of cervical samples (144). Among participants in the follow-up study, lower schooling and never using a condom were significantly higher in multivariate analysis (OR 1.94, 95% CI 1.2-3.2 and OR 2.00, 95% CI 1.0-3.9 respectively).

**Table 1:** Baseline characteristics associated with participation in follow-up study and with high-risk HPV infection outcome

Characteristic at enrollment in 2001	Category	Participation in follow-up study			HR-HPV infection outcome (Univariate)	
		**Enrolled 2001**	**Followed 2006**	**Multivariate**	**Type-specific Persistence**	**Incidence**

		(n = 969)*	(n = 576)*	***P *value**	OR (95% CI)**	OR (95% CI)***

Age	< 30 years old	23.4	19.8	0.813	Inf	**2.11 (1.17-3.80)**

Marital Status	Single/separated/widowed	24.4	20.7	0.169	0.18 (0.02-1.61)	0.40 (0.17-0.97)

Schooling	≤ 8 years	58.5	61.6	**0.011**	**5.00 (1.21-20.69)**	1.24 (0.70-2.19)

N° of Children	≥ 3	55.8	59.3	0.077	1.44 (0.43-4.85)	0.74 (0.43-1.28)

Age First Sexual Intercourse	≤16 years old	36.0	35.8	0.681	0.68 (0.21-2.17)	1.36 (0.79-2.36)

Lifetime Sexual Partners	> 1	39.6	36.0	0.701	0.74 (0.23-2.34)	0.78 (0.43-1.41)

Current High-risk Sexual Partner^a^	Yes	31.0	29.8	0.311	1.93 (0.49-7.61)	0.60 (0.27-1.35)

Condom Use	Never	84.0	85.5	**0.045**	0.18 (0.02-2.16)	0.90 (0.41-2.01)

History of STD^b^	Yes	3.8	4.2	0.222	1.88 (0.11-32.01)	0.36 (0.05-2.75)

Current Hormonal Contraception	Yes	12.0	13.3	0.733	0.39 (0.10-1.54)	1.33 (0.75-2.38)

Current Smoker	Yes	40.5	38.5	0.427	0.47 (0.14-1.55)	0.74 (0.42-1.31)

Previous Pap Test	No	21.2	17.0	0.7	14.35 (0.78-262.64)	**1.97 (1.06-3.67)**

Abnormal Baseline Pap^c^	Yes	3.8	3.7	0.904	0.32 (0.06-1.68)	0.92 (0.21-4.05)

Baseline HPV Status	HR-HPV positive	9.6	8.9	0.999	--	3.56 (1.77-7.16)

* values presented as percentages.

** referent: women who in 2006 had cleared a HR-HPV genotype present in 2001.

*** referent: women who did not acquire a new HR-HPV genotype in 2006.

^a ^partner had sex with other women during their relationship or ever had sex with prostitute.

^b ^sexually transmitted disease.

^c ^ASCUS or worse.

Bold: statistically significant at P value < 0.05.

Inf: infinite odds ratio since the characteristic was present in 0% of cases; thus the CI is incalculable.

The HR-HPV infections identified in the participants at baseline and follow-up are presented in Table 2. HR-HPV infection increased by 43%, from 8.8% to 12.7%. HPV16 prevalence did not change markedly in the period (2.1% - 3.3%); the largest increase of a particular HR-HPV genotype was for HPV18 (0.5% to 2.8%), which became the second most common HR-HPV in 2006 (fifth in 2001).

**Table 2:** High-risk HPV infections in a cohort of 576 women - Santiago, Chile

HR-HPV type	2001		2006		Prevalence Rate Ratio	
	**women**	**%**	**women**	**%**	**2006/2001**	**95% CI**

Any HR-HPV	51	8.8	73	12.7	1.4	**1.02 - 2.01**

HPV 16	12	2.1	19	3.3	1.6	0.78 - 3.23

HPV 18	3	0.5	16	2.8	5.3	**1.56 - 18.20**

HPV 31	8	1.4	5	0.9	0.6	0.21 - 1.90

HPV 33	2	0.4	1	0.2	0.5	0.05 - 5.50

HPV 35	2	0.4	3	0.5	1.5	0.25 - 8.94

HPV 39	4	0.7	8	1.4	2.0	0.61 - 6.61

HPV 45	3	0.5	10	1.7	3.3	0.92 - 12.05

HPV 51	1	0.2	5	0.9	5.0	0.59 - 42.67

HPV 52	2	0.4	6	1.0	3.0	0.61 - 14.80

HPV 56	8	1.4	11	1.9	1.4	0.56 - 3.39

HPV 58	7	1.2	4	0.7	0.6	0.17 - 1.94

HPV 59	4	0.7	2	0.4	0.5	0.09 - 2.72

HPV 66	2	0.4	2	0.4	1.0	0.14 - 7.08

HPV 68	1	0.2	0	0.0	0.0	--

Bold: statistically significant.

Prevalence, incidence and persistence of HR-HPV infections according to baseline age groups are presented in Table 3. In both years, HPV prevalence followed a U-curve, being highest at ages under 20 years (13.9% - 19.4%) and over 70 years (16.7% - 16.7%). Incidence was highest in women under 20 years old (19.4%) and lowest in women over 70 years old (0%). Incidence of new HR-HPV among women HR-HPV positive at baseline was three times higher (25.5%) than among women HPV negative at baseline (8.3%) (OR 3.8, 95% CI 1.8-8.0). In univariate analysis, baseline characteristics significantly associated with acquiring new HR-HPV types (*n *= 59), using women who did not acquire a HR-HPV (*n *= 517) as referent, were being younger than 30 years and not having a previous Pap test (Table 1); only the latter remained significant in multivariate analysis (OR 0.36, 95% CI 0.15-0.88). Some behavioral changes in the study period were associated with acquiring a new HR-HPV, however they did not reach statistical significance: becoming single or separated (OR 1.61, 95% CI 0.75-3.45), having new sexual partners (OR 1.12, 95% CI 0.46-2.74), having a new high-risk sexual partner (OR 1.38, 95% CI 0.50-3.77) and beginning smoking (OR 1.63, 95% CI 0.54-4.89).

**Table 3:** High-risk HPV status after 5 years in a cohort of 576 women - Santiago, Chile

Age in 2001 years	Number of women	Prevalence		Incidence	Persistence			
		**2001** ***n *(%)**	**2006** ***n *(%)**	***n *(%)**	**any type**		**type-specific**	

					***n *(%)**	**% of cohort**	***n *(%)**	**% of cohort**

≤ 20	36	5 (13.9)	7 (19.4)	7 (19.4)	0 (0.0)	0.0	0 (0.0)	0.0

21 - 30	84	8 (9.5)	12 (14.3)	12 (14.3)	2 (25.0)	2.4	0 (0.0)	0.0

31 - 40	112	9 (8.0)	9 (8.0)*	7 (6.2)	4 (44.4)	3.6	3 (33.3)	2.7

41 - 50	157	9 (5.7)	21 (13.4)	17 (10.8)	6 (66.7)	3.8	4 (44.4)	2.5

51 - 60	111	12 (10.8)	14 (12.6)*	11 (9.9)	7 (58.3)	6.3	5 (41.7)	4.5

61 - 70	58	5 (8.6)	7 (12.1)*	5 (8.6)	5 (100)	8.6	3 (60.0)	5.2

> 70	18	3 (16.7)	3 (16.7)	0 (0.0)	3 (100)	16.7	3 (100)	16.7

All	576	51 (8.9)	73 (12.7)*	59 (10.2)	27 (52.9)	4.7	18 (35.3)	3.1

Prevalence: women testing positive for one or more high-risk HPV genotypes in a given year.

Incidence: women with one or more newly detected high-risk HPV genotypes in 2006.

Persistence, any type: women testing positive for one or more high-risk HPV genotypes in both 2001 and 2006, regardless of genotype.

Persistence, type-specific: women testing positive for the same high-risk HPV genotype(s) in both 2001 and 2006.

* The number of incident cases plus the number of type-specific persistent cases does not equal the number of prevalent cases because some women have both a persistent high-risk HPV genotype and a newly detected one.

Of the 576 participants, 27 (4.7%) tested positive for any HR-HPV (regardless of type) both years and 427 (74.1%) were HPV negative both years. In univariate analysis, baseline characteristics associated with non-type-specific HR-HPV persistence, using women who remained HR-HPV negative as referent, were low schooling (OR 2.76, 95% CI 1.02-7.43) and an abnormal baseline Pap test (OR 12.55, 95% CI 3.30-47.71); however, only the later remained significant in multivariate analysis (OR 10.73, 95% CI 2.75-41.95).

HR-HPV type-specific persistence was 35.3%. All type-specific persistence occurred in women older than 30 years of age, increasing from 33.3% in women 31-40 years old to 100% in women over 70 years old. Additionally, a significant association with type-specific HR-HPV persistence *(n *= 18), in comparison with women who cleared a HR-HPV infection (*n *= 33), was observed with low schooling (Table 1); however, this did not reach statistical significance in multivariate analysis. We did not identify significant differences in persistence rates among different HR-HPV types: 5 out of 15 (33.3%) HPV 16/18 infections persisted, compared with 13 out of 44 (29.5%) other HR-HPV infections.

## Discussion

Reasons for the increase in HR-HPV infection are unclear, especially since the increase in age of the study population should have produced a decline in the number of HR-HPV positive women. The same protocol for HPV testing was used in both surveys and therefore the increase in HPV prevalence could not be attributed to an increase in sensitivity of HPV testing, but rather to an increase in exposure to HPV. A possible explanation for this is a change in sexual behavior; national health surveys have shown an increasing lifetime number of sexual partners among Chilean women [5]. In our cohort, women who acquired new HR-HPV infections had a greater increase in high-risk sexual behaviors than those who did not, though this difference did not reach statistical significance. The observed increase in HR-HPV infections suggests that a close surveillance of these infections and their associated lesions should be considered.

The age distribution of new infections showed a first peak in the youngest and a second peak at age 45 to 55 years old at follow-up. This second peak has been previously described in other Latin American countries [6]; however its significance is unclear, as it could represent newly acquired infections or dormant infections that have resurfaced in relation to menopausal changes. Based on the first hypothesis, some have recommended vaccination for adult women [7], while the second hypothesis suggests a more conservative approach. Answering this question will require more mechanistic research.

HR-HPV type-specific persistence increased with age, which is in line with findings reported in other studies [8,9]. Our finding that all HR-HPV infection cleared in women under age 30 indicates that the current recommendation of HPV screening for women over 30 years of age applies in our population [10,11].

Women with type-specific HR-HPV persistence are at high risk of developing cervical cancer [12-15] and should be under tight surveillance for precancerous lesions; they represent 3.1% (18 out of 576) of our study population. Our results indicate that if a screening program were implemented using a test like Hybrid Capture 2, which does not identify specific types of HR-HPV, 4.7% of our population would be called for further work up, thus including the 1.6% of women who in fact did not persist with the same HR-HPV type. This possibly unnecessary follow-up could be avoided if a type-specific test were used, but this should be balanced against the higher cost of the test. Recent studies conducted in Canada [16], Mexico [17], South Africa [18], Europe and the US [19] have shown that implementation of an HPV screening strategy, even without genotyping, can be highly cost effective when compared with cytology screening alone.

A major strength of this study is that it is the first in Chile that follows a population-based cohort of women to learn about the natural history of HR-HPV infection in our population. Other strengths include the broad age range of enrolled women, the collection of comprehensive information on risk factors, and the performance of DNA testing at a world reference laboratory.

In conclusion, HR-HPV prevalence in our study population increased during the follow-up period, presumably the result of changes in sexual behavior, which could lead to an increase in cervical cancer in this population. In order to strengthen our prevention programs, newly available tools such as HPV-based screening tests should be considered in Chile. In our study, all HR-HPV infections in women under 30 years old cleared, supporting the current recommendation of HR-HPV screening for women over 30 years of age.

## Abbreviations

HPV: human papillomavirus; HR: high-risk; ASCUS: atypical squamous cells of undetermined significance; STD: sexually transmitted disease; OR: odds ratio; CI: confidence interval.

## Competing interests

The authors declare that they have no competing interests.

## Authors' contributions

CF was responsible for the study conception, design and implementation, and analysis and interpretation of data. FO carried out the field work and was responsible for data collection. KP coordinated the field work and AC coordinated the collection and preparation of samples at the laboratory. AD performed the statistical analyses. CF, VV, SF and PS were responsible for the analysis and interpretation of data. CF, VV and AD drafted the manuscript. All authors revised the manuscript critically and approved the final version for publication.
